# Effect of tooth preparation design on fracture load and fracture behavior of deciduous crowns fabricated by subtractive and additive manufacturing

**DOI:** 10.1007/s00784-026-07127-9

**Published:** 2026-09-16

**Authors:** Misaki Hamashima, Kanae Wada, Shinya Oishi, Hidekazu Takahashi, Kaori Kohi, Daiki Kondo, Yujeong Shin, Yuika Yamanaka, Atsushi Oishi, Asuna Sugimoto, Kanako Kishima, Yumiko Nakashima, Rika Kurogoshi, Tsutomu Iwamoto

**Affiliations:** 1 Department of Pediatric Dentistry/Special Needs Dentistry, Division of Oral Health Sciences, Graduate School of Medical and Dental Sciences, Institute of Science Tokyo, 1-5-45 Yushima, Bunkyo-ku, Tokyo Japan; 2 Department of Oral and Maxillofacial Rehabilitation, Prosthodontics, Institute of Science Tokyo, Tokyo, Japan; 3 Department of Oral Biomedical Engineering, Graduate School of Medical and Dental Sciences, Institute of Science Tokyo, Tokyo, Japan

**Keywords:** CAD-CAM, Subtractive-manufacturing, Additive manufacturing, Deciduous crowns, Fracture load, Fracture behavior

## Abstract

**Objectives:**

This study evaluated the effects of crown material and margin design on fracture load and behavior of computer-aided design/computer-aided manufacturing (CAD-CAM) crowns for deciduous molars.

**Methods:**

Three subtractive-manufacturing (SM) materials and two additive-manufacturing (AM) materials were evaluated. Crowns with knife-edge, chamfer, and deep chamfer margins were fabricated and bonded to standardized deciduous molar abutments (*n* = 12/group; total = 180). Fracture load was measured under static loading, and fracture modes were classified by repairability and abutment involvement. Fracture surfaces and crack propagation were examined by scanning electron microscopy.

**Results:**

Significant effects of material and material–margin interactions were observed (*p* < 0.05). ESTELITE BLOCK II exhibited the highest fracture load with knife-edge margins, whereas Detax showed the highest values with deep chamfer margins. However, non-repairable abutment-involving fractures occurred in both materials with knife-edge margins. CERASMART PRIME showed lower fracture load but mainly repairable fractures. SHOFU BLOCK HC and CERASMART PRIME, unified crown–abutment fracture in ESTELITE BLOCK II, interlayer delamination in Detax, and superficial radial cracking in NextDent.

**Conclusions:**

The effects of margin design on fracture load and fracture behavior were material-dependent. Among subtractive-manufacturing materials, fracture load was generally consistent with reported mechanical properties, whereas fracture behavior in additive-manufacturing materials may have been influenced by internal structure and build orientation. Material selection should consider fracture load, fracture severity, and abutment involvement.

**Clinical relevance:**

Material and margin selection influence fracture resistance and failure patterns. Fracture severity and abutment involvement should be considered in addition to fracture load.

## Introduction

Deciduous teeth are not merely temporary substitutes for permanent teeth; they play essential roles in craniofacial development and maintaining masticatory, phonatory, and respiratory functions. Therefore, preserving and appropriately restoring deciduous teeth are critical in pediatric dental practice.

For deciduous molars, crown restoration is widely applied following endodontic treatment or multi-surface caries with substantial tooth structure loss. Such restorations must maintain function for approximately 5–7 years until natural exfoliation.

Stainless steel crowns (SSCs) have long been used for coronal restoration of deciduous teeth. SSCs provide high durability, ease of handling, and well-documented long-term clinical performance [1]. However, increasing esthetic demands have led to growing interest in tooth-colored restorative materials.

Prefabricated zirconia crowns (PZCs) have become increasingly popular due to these demands. Although PZCs exhibit excellent fracture and wear resistance, they have clinical limitations, including difficulty achieving optimal fit and the need for extensive tooth preparation [2]. Moreover, their high hardness [3] may cause wear of opposing enamel [4, 5]. Their limited adaptability to physiological attrition and occlusal changes associated with growth may further restrict their use in pediatric dentistry [4, 5].

Resin-based crowns fabricated using computer-aided design/computer-aided manufacturing (CAD-CAM), including subtractive manufacturing (SM) and additive manufacturing (AM), have recently gained attention. Resin-based materials provide superior esthetics and machinability and cause less wear on opposing teeth than zirconia [5]. Moritz et al. reported that CAD-CAM hybrid resin crowns exhibited sufficient fracture resistance to withstand masticatory forces in deciduous teeth [6]. Shin et al. demonstrated that certain AM resin materials showed wear properties comparable to deciduous enamel [7]. Yamanaka et al. suggested that stress transmission to the abutment tooth varies with crown material, indicating that material properties influence the mechanical behavior of restored teeth [5]. Wada et al. [21] also reported favorable wear characteristics between resin-based materials and deciduous enamel, supporting their applicability as restorative materials [8, 9].

Deciduous teeth differ anatomically from permanent teeth, with thinner enamel and dentin and a smaller tooth structure volume [10]. Therefore, preparation guidelines for CAD-CAM crowns in permanent teeth cannot be directly applied to deciduous teeth [11, 12]. Although CAD-CAM crowns provide excellent fit, tooth reduction is required to ensure marginal accuracy [13]. Excessive preparation increases the risk of pulp exposure and structural fracture, emphasizing the need for appropriate preparation designs.

In addition to material properties, margin configuration influences restoration durability by affecting stress concentration and load distribution. However, studies investigating interactions between material properties and margin configuration in CAD-CAM crowns for deciduous teeth remain limited [14, 15]. Previous studies have mainly focused on material mechanical properties, with insufficient evaluation of preparation designs and fracture modes.

Furthermore, occlusal forces in children are lower than those in adults and increase from the deciduous to mixed dentition stages [16]. Therefore, evaluating deciduous crowns requires consideration of not only material load but also the combined effects of material properties and tooth preparation on fracture behavior.

Accordingly, this study aimed to fabricate deciduous crowns using CAD-CAM materials and evaluate fracture load and fracture modes under different margin configurations. This approach aimed to clarify the relationship between material properties and tooth preparation and propose optimal preparation guidelines for deciduous tooth crowns. The null hypothesis was that neither crown type nor margin design would affect the fracture load, and that no interaction would exist between these factors.

## Materials and methods

This study evaluated the effect of margin design on the fracture load and behavior of subtractive- and additive-manufactured crowns in deciduous teeth. Fracture origin, crack propagation, and fracture patterns were further characterized using scanning electron microscopy (SEM).

### Preparation of the abutment tooth

Artificial mandibular left first deciduous molars (B4-309B, #75; Nissin Dental Products Inc., Kyoto, Japan) were prepared using diamond burs. Three marginal designs with different thicknesses were prepared: knife-edge (0.5 mm), chamfer (1.1 mm), and deep chamfer (1.5 mm) (Table 1). Knife-edge preparation followed the conventional design for preformed metal crowns in deciduous teeth. Occlusal reduction was standardized at 0.5 mm for all specimens [15]. All preparations were performed by a single operator under water cooling using diamond burs (301, FG102R: working tip diameter 1.1 mm; 109R: 1.5 mm; SHOFU Inc., Kyoto, Japan).

**Table 1.**
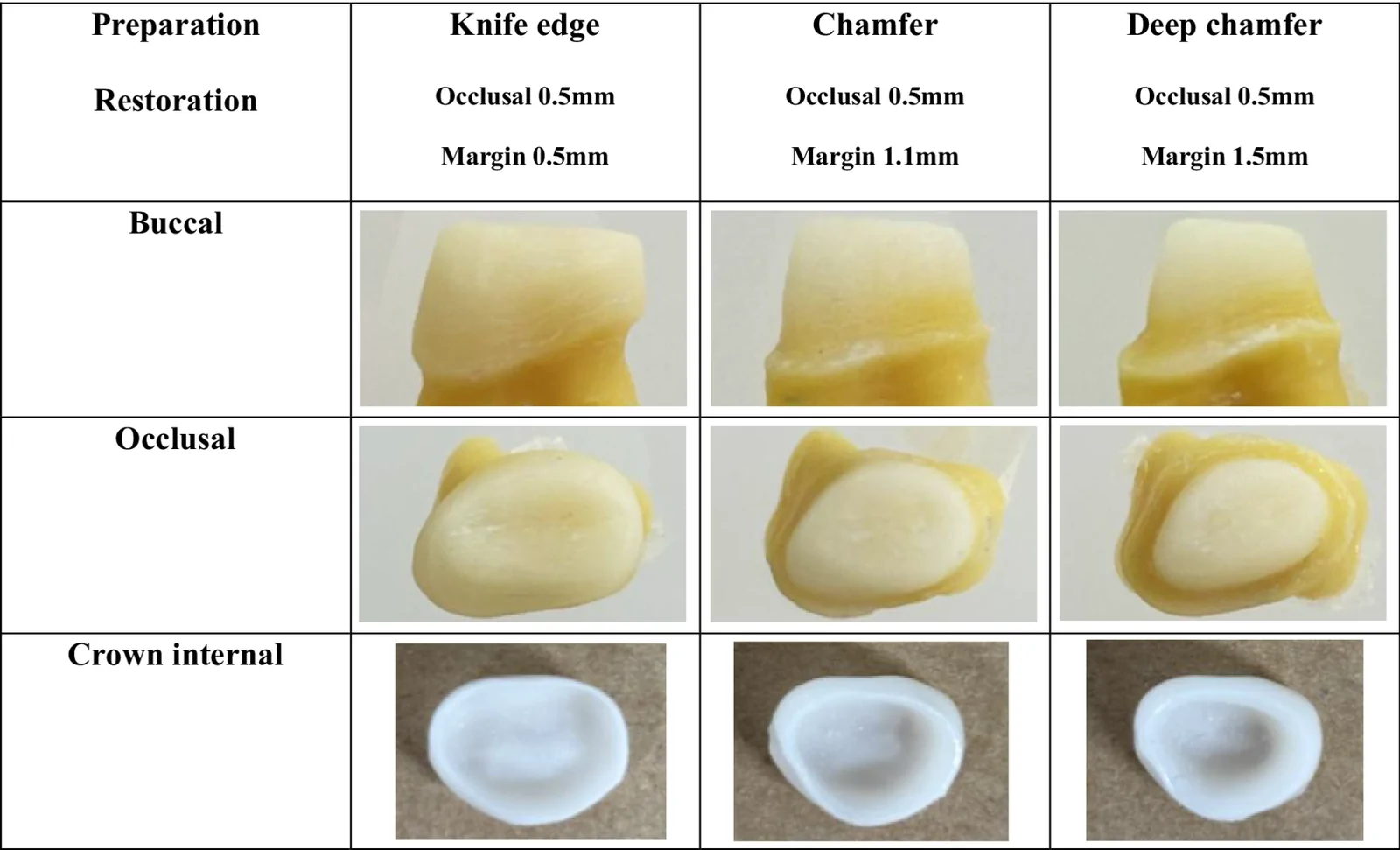
Marginal configurations of the three types of abutment teeth in this study

The marginal and occlusal surfaces of the abutment teeth were viewed, and the inner surfaces of the crowns corresponded to each marginal configuration

Prepared abutment teeth were duplicated using a transparent silicone impression material (KE-1606; Shin-Etsu Chemical Co., Gunma, Japan). A fiber-reinforced flowable resin (everX Flow^®^, GC Corporation, Tokyo, Japan), with an elastic modulus of 9.0 GPa [17], close to that of deciduous dentin (11.59–16.91 GPa) [18], was injected into the molds and light-cured to fabricate replicated specimens according to Oishi et al. [19].

For each margin design, 12 specimens (*N* = 12) were fabricated for each of the five materials using silicone molds, resulting in 180 abutment teeth. Sample size was calculated a priori using G*Power 3.1. Assuming α = 0.05, power = 0.80, and an effect size from a previous fracture resistance study [20], at least 10–12 specimens per group were required.

Crown data were generated using the double-scan method described by Yamanaka et al. [5]. The artificial tooth before preparation and the prepared abutment tooth were scanned separately using a desktop scanner (AutoScan-DS-EX Pro; Shining 3D Dental, Hangzhou, China), and the datasets were superimposed to obtain the final crown data.

### Crown types and fabrication methods

In this study, five CAD-CAM resin-based materials were evaluated, comprising three subtractive manufacturing (SM) materials and two additive manufacturing (AM) materials. All materials contained filler particles. The SM resin materials all contained urethane dimethacrylate (UDMA) and were selected based on differences in filler morphology. The AM resin materials were selected to represent two different flexural load properties. Three CAD-CAM resin-based block materials were used for SM: SHOFU BLOCK HC (HC; SHOFU INC., Kyoto, Japan), CERASMART PRIME (CP; GC Corp., Tokyo, Japan), and ESTELITE BLOCK II (EB; Tokuyama Dental Corporation, Tokyo, Japan). Two AM materials for the vat-photopolymerization method were also included: Detax FREEPRINT^®^ Crown(DT; detax, Ettlingen, Germany) and NextDent^®^ DH Print Provisional CB(ND; NextDent B.V., Soesterberg, Netherlands), for a total of five materials (Table 2).

**Table 2:** Summary of crown materials used in this study

Group	Abbreviation	Material	Composition	Manufacture
SM	HC	SHOFU BLOCK HC	UDMA, TEGDMA, Silica	SHOFU INC., Kyoto, Japan
	CP	CERASMART PRIME	UDMA, Silica(20 nm), barium glass	GC Corp., Tokyo, Japan
	EB	ESTELITE BLOCK Ⅱ	UDMA, TEGDMA, Silica, Silica Zr filler	Tokuyama Dental Corp., Tokyo, Japan
AM	DT	Detax FREEPRINT Crown	45 < 60wt% IsopropylidenediphenolPeg-2 Dimethacrylat,1 < 5wt% 2Hydroxyethylmethacrylat, 1 < 5wt% Diphenyl (2,4,6 trimethylbenzoyl) phosphinoxid,1-< 5wt% Hydroxypropylmethacrylat, < 1wt% Phenyl-bis (2,4,6-trimethylbenzoyl) - phosphinoxid	detax, Ettlingen, Germany
	ND	NextDent DH Print Provisional CB	> 90%methacrylic oligomers, methacrylate monomer, < 3%phosphine oxides, pigment	NextDent B.V., Soesterberg, Netherlands

DT was selected based on a previous study by Shin et al., reporting a relatively smooth worn surface after wear against deciduous enamel [7]. CP was selected because favorable results have been reported in strain tests evaluating combinations of deciduous crowns and core materials [5].

Crown design data were generated from a conventionally used preformed metal crown (3 M stainless-steel deciduous molar crown; 3 M, Beirut, Lebanon). The crowns were scanned using a desktop scanner (AutoScan-DS-EX Pro; Shining 3D Dental, Hangzhou, China), and the scanned data were used to construct the crown dataset.

CAD-CAM block materials were milled using an MD-500 unit (Canon Electronics Inc., Saitama, Japan), whereas AM materials were fabricated by vat photopolymerization (Detax: Rapid Shape GmbH, Heimsheim, Germany; NextDent: NextDent 5100). For all AM crowns, the build orientation was fixed and set perpendicular to the loading direction.

For each of the five materials, 36 crowns were fabricated, yielding 180 deciduous crowns. The cement space was set at 80 μm for all crowns.

The fabricated crowns were bonded to the abutment teeth according to the manufacturers’ instructions. Before bonding, the crown intaglio surfaces and abutment teeth were pretreated.

The crown intaglio surfaces were sandblasted with 70 μm Al₂O₃ at 0.2 MPa for 10 s from a distance of 10 mm, followed by silane treatment using a ceramic primer (CLEARFIL^®^ CERAMIC PRIMER PLUS; Kuraray Noritake Dental Inc., Tokyo, Japan). The abutment teeth were etched with 40% phosphoric acid gel (K-ETCHANT GEL; Kuraray Noritake Dental Inc., Tokyo, Japan) for 5 s, rinsed, air-dried, treated with the same ceramic primer, and primed with PANAVIA^®^ V5 Tooth Primer (Kuraray Noritake Dental Inc., Tokyo, Japan).

After pretreatment, the crowns were cemented using resin cement (PANAVIA^®^ V5; Kuraray Noritake Dental Inc., Tokyo, Japan). Light curing was performed for 20 s from the buccal, lingual, and occlusal aspects using a light-curing unit (PenCure 2000; Morita Corp., Osaka, Japan) with an output intensity of 1000 mW/cm².

### Fracture testing and observation of fracture modes

After bonding, specimens were embedded in the center of an aluminum tube (20 mm in diameter) using autopolymerizing resin (Palapress^®^ vario; Kulzer GmbH, Hanau, Germany), leaving 1 mm of tooth structure below the cementoenamel junction exposed.

**Fig. 1:**
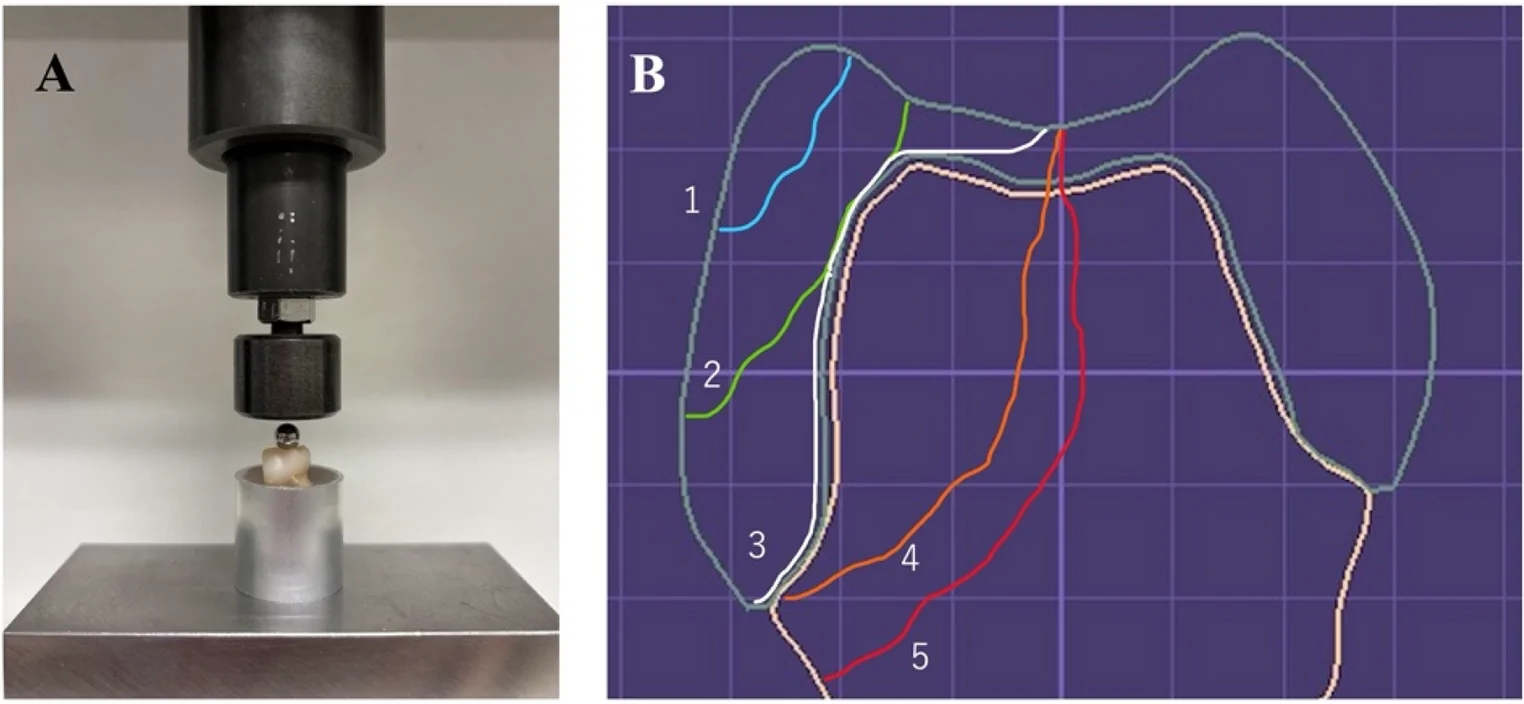
Universal testing machine used and a schematic diagram of the classification of five failure modes (**A**): To simulate bone levels, the specimens were embedded in methacrylate resin at a position 1 mm from the cementoenamel junction (CEJ), and static load testing was performed on all specimens using a universal testing machine. (**B**): CEJ, cemento–enamel junction. (1) chipping failure, (2) repairable unilateral interfacial fracture without abutment tooth involvement, (3) repairable bilateral interfacial fracture without abutment tooth involvement, (4) fracture involving the abutment tooth within a repairable level, and (5) fracture involving the abutment tooth within a non-repairable level

Fracture testing was performed using a universal testing machine (EZ-SX; Shimadzu Corporation, Kyoto, Japan). To simulate clinically relevant loading conditions and avoid excessive stress concentration, a stainless-steel ball (5 mm in diameter) was used to apply the load to the inner inclines of the cusps and central fossa of the mandibular deciduous molar (Fig. 1), as previously described [20, 22]. 

A vertical load was applied at a crosshead speed of 1.0 mm/min until fracture occurred. The load at failure was recorded as the fracture load (N).

Fracture modes were classified by two examiners according to the predefined classification criteria described below. The examiners were blinded to the material and tooth preparation design during fracture classification. Fractures extending > 1 mm apical to the cemento-enamel junction (CEJ) were classified as non-restorable [23], whereas the others were considered restorable. Fracture outcomes were classified into five categories: (1) chipping failure, (2) repairable unilateral interfacial fracture without abutment tooth involvement, (3) repairable bilateral interfacial fracture without abutment tooth involvement, (4) fracture involving the abutment tooth within a repairable level, and (5) fracture involving the abutment tooth within a non-repairable level. Fracture modes are shown on the right side of Fig. 1. A total of 180 specimens were allocated to five material groups and three preparation designs, with 12 specimens in each group (*n* = 12).

### Scanning electron microscope observation

The fractured crown surfaces were examined using a field-emission scanning electron microscope (SEM) (JSM-7900 F; JEOL, Tokyo, Japan) at 15 kV. Low-magnification images were acquired using the Long Depth of Focus (LDF) mode. The specimens were coated with a thin layer of platinum using a sputter coater (Desk II, Denton Vacuum, Moorestown, NJ, USA) to provide conductivity.

### Statistical analysis

Statistical analyses were performed using the IBM SPSS Statistics Base version 31 (IBM Corp., Armonk, NY, USA). The normality of the data distribution was assessed using the Shapiro–Wilk test. Two-way analysis of variance (ANOVA) was performed to evaluate the effects of crown type (HC, CP, EB, DT, and ND) and margin design (knife-edge, chamfer, and deep chamfer) on the fracture load. When a significant interaction was detected, simple main effect analyses were conducted, followed by Bonferroni-adjusted pairwise comparisons. Statistical significance was set at *p* < 0.05.

## Results

**Fig. 2:**
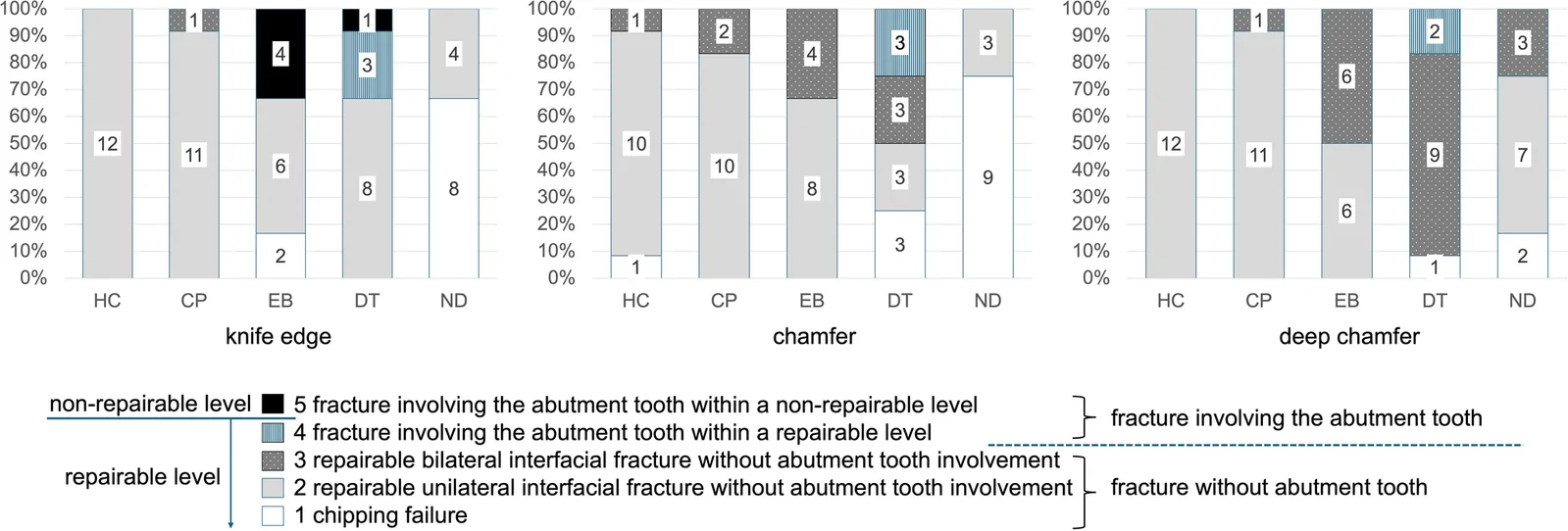
Comparison of fracture modes according to crown type and margin configuration. The number of fractures in each mode is presented for each group. Specimens in the HC and CP groups predominantly exhibited repairable unilateral fractures across all marginal configurations, indicating stable fracture patterns. EB showed more severe fracture modes in the knife-edge margin; however, the fracture severity decreased with increasing marginal thickness. DT demonstrated margin-dependent variations in fracture mode, with increasing marginal thickness associated with more diverse fracture patterns, including abutment fractures. ND predominantly exhibited minor fractures, although fracture severity increased with increasing marginal thickness

Figure 2 shows the fracture modes according to crown type and margin configuration. In the SM group, category 2 was most frequently observed for both HC and CP across all margin configurations (HC category 2: knife-edge 100%, chamfer 83.3%, deep chamfer 100%; CP category 2: knife-edge 91.6%, chamfer 83.3%, deep chamfer 91.6%), indicating predominantly repairable unilateral fractures.

For EB, category 2 (50%) and non-repairable fracture category 5 (33.3%) were observed in the knife-edge margin. In the chamfer margin, categories 2 (66.6%) and 3 (33.3%) were predominant, while categories 2 (50%) and 3 (50%) were most frequent in the deep chamfer margin. These findings suggest decreased fracture severity with increasing marginal thickness.

In the AM group, DT showed the highest proportion of category 2 fractures in the knife-edge margin (66.6%), with repairable abutment-involving fractures (category 4) in 25% and non-repairable fractures (category 5) in 8.3% of cases. Categories 1–4 were evenly distributed (approximately 25% each) in the chamfer margin. In the deep chamfer margin, category 3 was predominant (75%), while category 4 was also observed (16.6%). Although fracture load increased with marginal thickness, fracture patterns became more diverse, including abutment-involving fractures.

For ND, category 1 fractures were predominant in the knife-edge and chamfer margins (66.6% and 75%, respectively), indicating frequent minor fractures. In the deep chamfer margin, category 2 fractures increased (58.3%), and category 3 fractures were also observed (25%); however, repairable fracture patterns remained predominant despite increased marginal thickness.

**Fig. 3:**
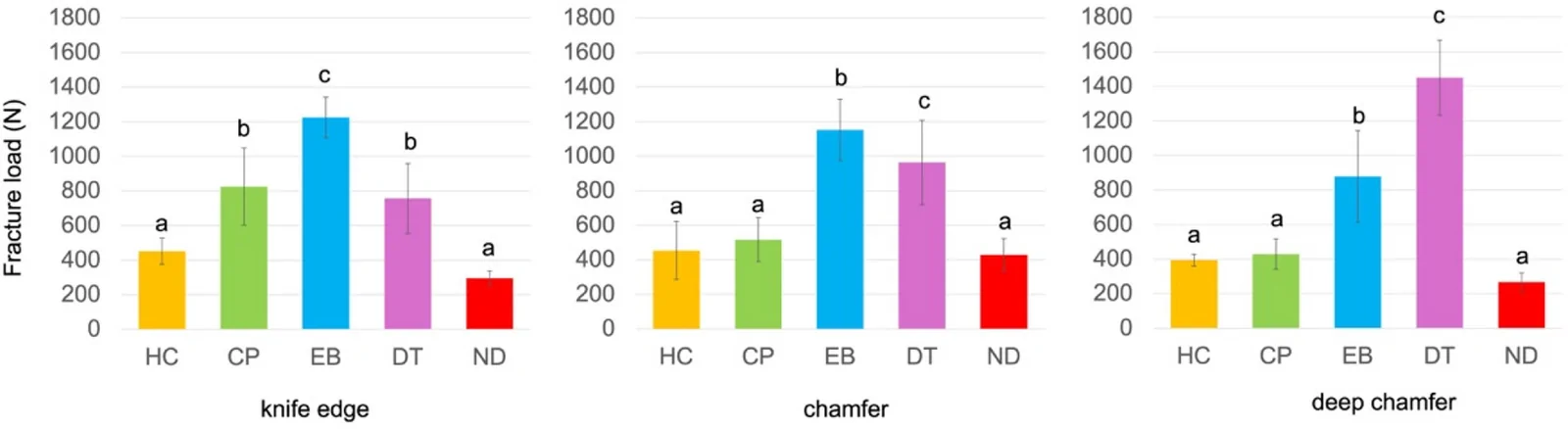
Effect of crown type within each margin configuration. Groups sharing the same letter were not significantly different. For the knife-edge margin, EB exhibited the highest fracture load among all groups, whereas ND showed the lowest values. For the chamfer margin, EB demonstrated significantly higher values than the other groups, and DT also showed significant differences compared to some groups. For the deep chamfer margin, both the DT and EB exhibited higher values, with the DT exhibiting the highest fracture load. In contrast, ND consistently demonstrated significantly lower values under all conditions

Figure 3 shows the effect of crown type within each margin design. A two-way ANOVA was performed to evaluate the effects of crown type (HC, CP, EB, DT, and ND) and margin configuration on fracture load. Crown type had a significant effect, whereas margin configuration did not. A significant interaction was observed between the two factors (*p* < 0.001). Simple main effects were therefore analyzed. Significant differences in crown type were found for all margin designs (Bonferroni’s test).

For the knife-edge margin, the EB group showed significantly higher fracture load than the other groups (*p* < 0.001).

For the chamfer margin, the EB group showed significantly higher fracture load than the DT (*p* = 0.045) and other groups (*p* < 0.001). No significant differences were observed among the HC, CP, and ND groups.

For the deep chamfer margin, the DT group showed significantly higher fracture load than the other groups (*p* < 0.001). No significant differences were observed among the HC, CP, and ND groups.

**Fig. 4:**
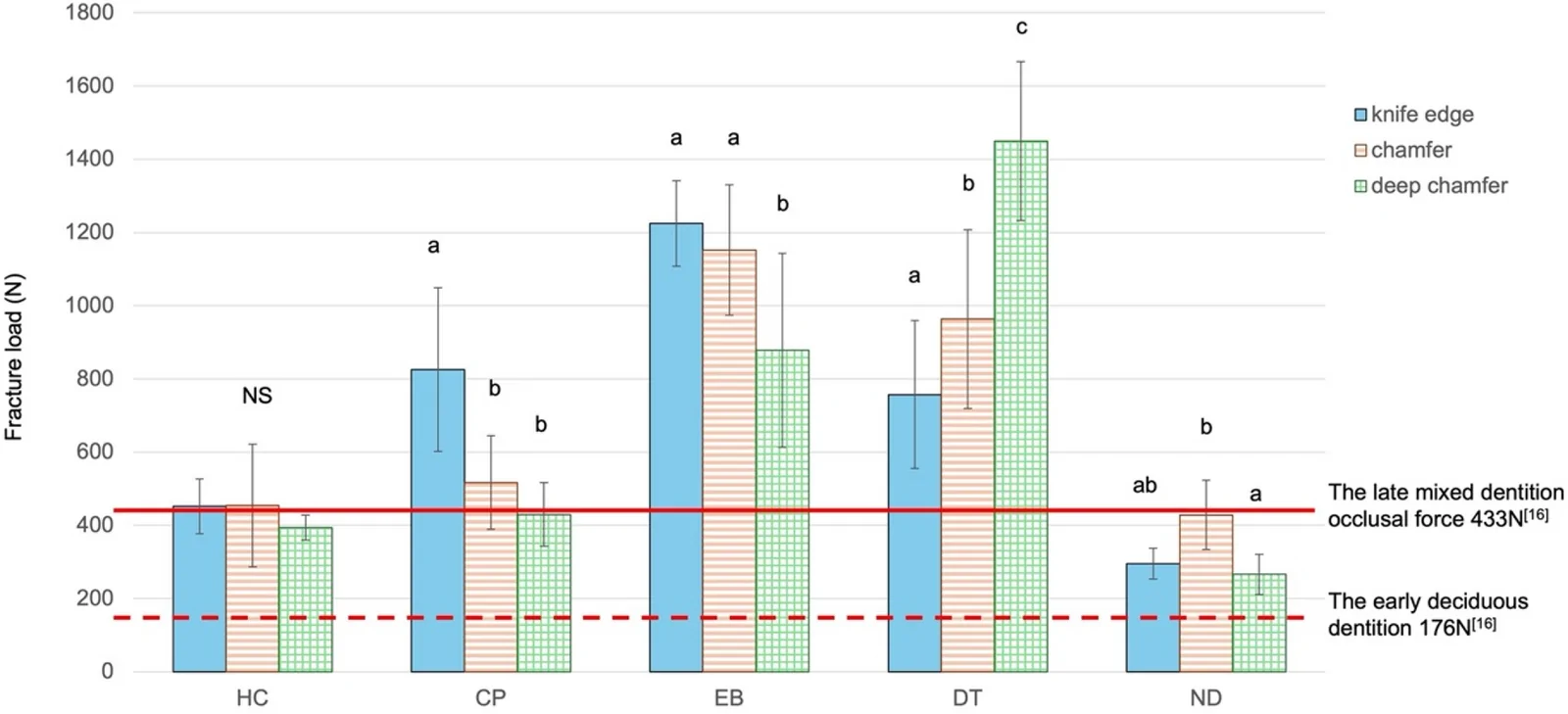
Effect of margin configuration within each crown type. Groups sharing the same letter were not significantly different. In the SM group, no significant differences were observed for HC; however, in the CP and EB groups, the fracture load decreased with increasing marginal thickness, with significant differences among the margin configurations. In the AM group, the DT group showed a significant increase in fracture load with increasing marginal thickness, whereas in the ND group, the chamfer margin was significantly higher than the deep chamfer margin

Figure 4 shows the effect of margin configuration for each crown type. The effect of margin configuration within each crown type was examined. In the SM group, no significant differences were observed among the margin configurations of HC. In contrast, CP and EB showed the highest fracture loads with the knife-edge margin and a decreasing trend toward the deep chamfer margin, with significant differences among margin designs (*p* < 0.001).

In the AM group, DT showed a significant increase in fracture load with increasing marginal thickness. Significant differences were found between the knife-edge and chamfer (*p* = 0.006), chamfer and deep chamfer (*p* < 0.001), and knife-edge and deep chamfer (*p* < 0.001). Conversely, ND showed a significantly higher fracture load for the chamfer margin than the deep chamfer margin (*p* < 0.042), although no consistent trend was observed.

**Fig. 5:**
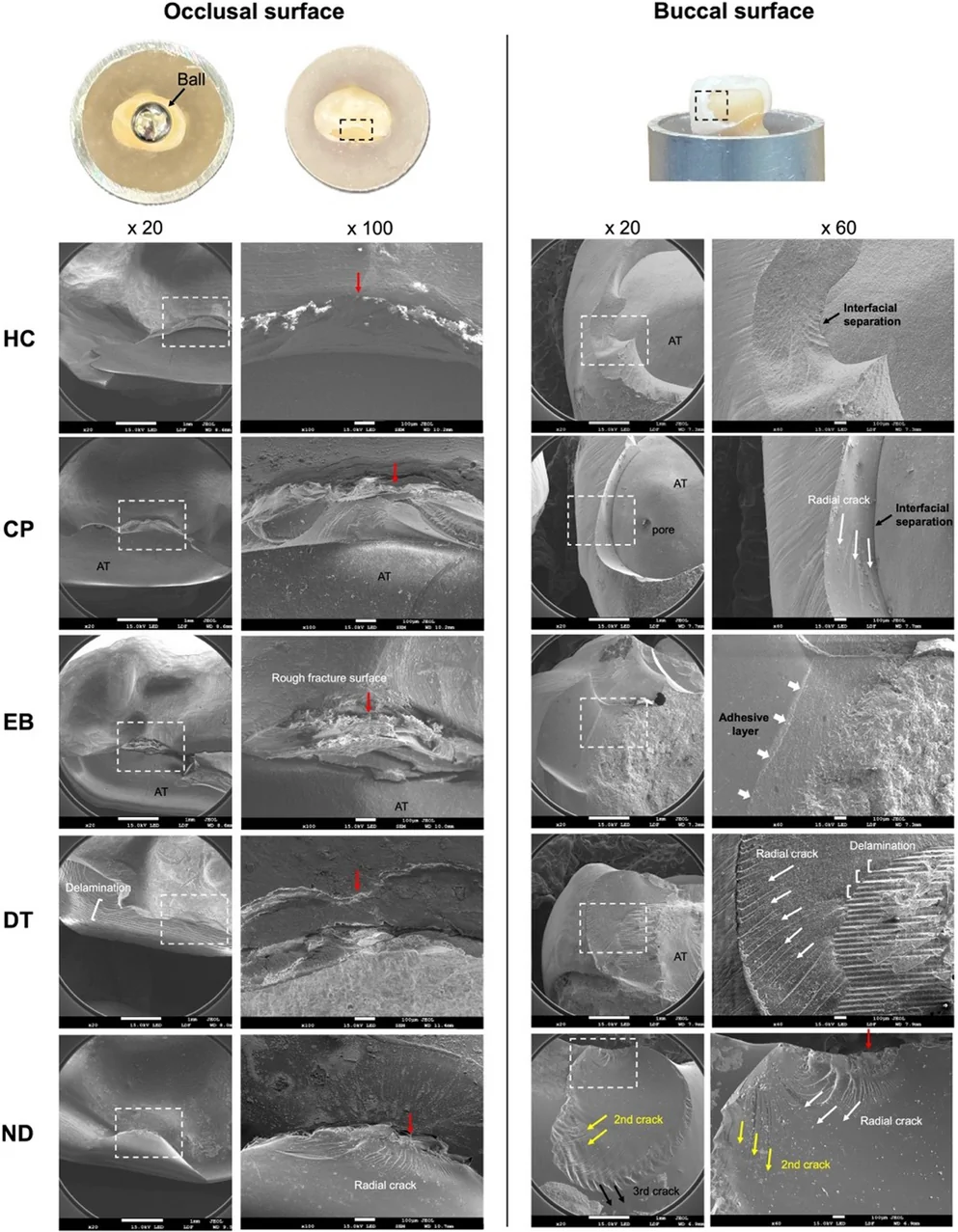
SEM images of CAD-CAM crown surfaces after fracture testing. The estimated origin of the fracture is indicated by a red arrow. Abutment tooth: AT. In HC, debonding is observed along the adhesive interfaces of the fractured surfaces. No radial cracks were observed in the crowns. In CP, debonding is observed along the adhesive interfaces of the fractured surfaces. Additionally, radial cracks propagating distally were identified within the crown of the cross section. Unlike the HC and CP groups, no evident debonding was observed at the adhesive interface on the fracture surface in the EB and DT groups. Radial cracks originating from the initiation site were observed within the crown. Radially oriented cracks propagating from the inner aspect of the crown toward the superficial layer were observed on the fractured surface. Moreover, a laminated crown structure was detected on the abutment side, indicating that interlayer delamination occurred within the crown. The fracture was confined to the superficial crown layer, where radially oriented cracks originating from the initiation site were observed. Secondary radial cracks associated with changes in the direction of stress propagation were identified away from the initiation site

Figure 5 shows SEM images. Among the SM materials, HC and CP exhibited debonding along the adhesive interface. In contrast, EB showed no evident debonding at the adhesive interface; instead, the crown and abutment appeared to fracture as a single unit with no observable gap at the interface. In the AM materials, the DT exhibited a fracture pattern suggestive of interlayer delamination, whereas the ND exhibited radially oriented cracks confined to the superficial layer.

## Discussion

This study evaluated the effects of crown type and margin design on fracture load and fracture modes in CAD-CAM deciduous crowns. The findings demonstrated that fracture load did not necessarily correspond to fracture severity or abutment involvement. Although EB in the SM group and DT in the AM group showed high fracture loads, catastrophic fractures involving the abutment tooth occurred depending on the margin design. Conversely, HC and CP in the SM group, despite showing relatively lower fracture loads, exhibited a greater tendency toward repairable fractures. These findings highlight that fracture load and fracture severity, including abutment involvement, should be considered as complementary endpoints when evaluating the mechanical performance of deciduous crowns. Thus, a higher fracture load does not necessarily indicate a more favorable failure pattern or better overall clinical performance. The null hypothesis was partially rejected because crown type significantly affected fracture load, and a significant interaction was observed between crown type and margin design, although the main effect of margin design was not significant.

Stresses on teeth and restorations are generally low and repetitive. Taha et al. reported that fracture resistance under static loading exceeds functional occlusal loads, supporting its use for comparing restorative materials and cavity designs [24]. Because fatigue and static loading are linearly related, compressive static testing provides information on fracture behavior and load-bearing capacity [25].

A fiber-reinforced flowable resin was used as the abutment material to approximate dentin elastic modulus and standardize stress distribution. Since natural deciduous teeth vary in morphology and mechanical properties, a standardized material was required. The elastic modulus of the abutment material influences crown fracture load and stress distribution at the adhesive interface [6]. Therefore, short-fiber-reinforced composite resin (everX Flow), which inhibits stress dispersion and crack propagation [26], was used. Its elastic modulus (9.0 GPa) [17] approximates that of deciduous dentin (11.59–16.91 GPa) [18]. Furthermore, the resin-based artificial abutment may provide a different bonding substrate from a prepared natural deciduous molar, where enamel reduction may result in a greater proportion of dentin being exposed at the bonding surface. This difference in substrate may influence the bonding characteristics and, consequently, the fracture load and fracture patterns observed in the present study.

Among SM materials, HC (elastic modulus: 10.4 GPa; flexural load: 132 MPa), CP (12.0–12.8 GPa; 174.2 MPa), and EB (13.8 GPa; 195 MPa) [6, 27, 28] showed differences in fracture load that were generally consistent with differences in their reported elastic modulus and flexural strength. In contrast, for the AM materials, although DT (elastic modulus: 2.5 GPa; flexural load: 98.81 MPa) and ND (2.3 GPa; 89.39 MPa) had similar reported mechanical properties [29, 30], differences in fracture load were observed.

Regarding the filler content, SM materials have been reported to contain 61–68 wt% for HC, 71 wt% for CP, and 70 wt% for EB [28]. In contrast, among the AM materials, DT contains 23 area% filler with a wide particle size distribution (0.14–5.71 μm) [31], whereas ND has been reported to contain 1–20 wt% inorganic fillers, including Al, Si, Ti, and Fe [32]. The UDMA-based matrix used in SM materials generally allows higher filler loading than bis-GMA-based systems due to its lower viscosity and higher reactivity. However, despite similar reported filler contents in CP and EB, differences in fracture load were observed, suggesting that fracture load cannot be explained solely by filler content.

Yamanaka et al. reported that greater strain was observed in deciduous teeth restored with resin-based crowns than in those restored with zirconia crowns, suggesting that stress transmission differs depending on the material [5]. In the present study, the EB and DT, which exhibited high fracture loads, showed fractures extending to the abutment tooth in the knife-edge margin design, whereas the ND showed relatively minor fracture patterns. These findings suggest that fracture behavior may be influenced not only by mechanical properties but also by matrix composition, particle size distribution, and stress transmission characteristics.

SEM observations revealed that among the SM materials, HC and CP exhibited crack propagation and interfacial failure, whereas among the AM materials, DT showed delamination-like fractures, and ND demonstrated surface-limited radial cracks. These findings suggest that, in contrast to the relatively homogeneous structure of SM materials [33], the layered structure of AM materials may have contributed to differences in fracture propagation behavior [34]. Because all AM crowns in the present study were fabricated using a fixed build orientation perpendicular to the loading direction, the observed fracture behavior may also reflect the orientation-dependent characteristics of the printed materials. However, anisotropy and interlayer bond strength were not directly evaluated in the present study, and therefore the contribution of these factors to the observed fracture patterns remains uncertain. In addition, crack initiation and propagation may also have been influenced by the homogeneous structure and mechanical properties of the artificial abutment used in the present study.

Furthermore, the differences in fracture behavior between the DT and ND may be associated with variations in the composition of each AM resin matrix. The mechanical properties of resin-based materials are known to depend on the structure of the monomers and oligomers, as well as the degree of cross-linking density [35]. Low-molecular-weight and multifunctional monomers may increase cross-linking density but may also increase brittleness [35]. DT contains bisphenol A–derived dimethacrylate (isopropylidenediphenol PEG-2 dimethacrylate) as the major component, along with HEMA (2-hydroxyethyl methacrylate) and HPMA (hydroxypropyl methacrylate). This composition may contribute to improved stress distribution and higher fracture load compared with ND. In contrast, ND primarily consists of methacrylic oligomers and methacrylate monomers, and differences in resin matrix composition may potentially contribute to the surface-confined radial cracks observed in SEM images. However, the polymer network structure and cross-linking density were not directly evaluated in the present study. These findings suggest that the differences in fracture load and fracture patterns between the DT and ND may be associated with differences in resin matrix composition and layered structure. However, the relative contributions of these factors could not be determined in the present study. The DT demonstrated a notably high fracture load in the deep chamfer configuration (1449.8 N), closely approximating the previously reported fracture resistance of AM crowns (1495.05 N) [36]. Moreover, the fracture load increased with the marginal thickness, which may indicate a reinforcing effect related to the layered structure under the selected build orientation.

Adhesive bonding is known to influence the fracture load and mechanical behavior of restorations [37–39]. Finite element analysis (FEA) studies have demonstrated that materials with higher elastic moduli reduce stress distribution within the cement layer, thereby decreasing the likelihood of debonding [40, 41]. In contrast, materials with lower elastic moduli are more prone to high stress concentrations, which may increase the risk of interfacial failure [42]. In this study, the bonding conditions and luting agents were standardized; thus, the influence of adhesive factors was considered minimal. Nevertheless, scanning electron microscopy (SEM) revealed material-dependent differences in interfacial behavior. Interfacial debonding along the adhesive interface was observed in the HC and CP, whereas no distinct interfacial separation was detected in the EB. Instead, the EB exhibited a fracture mode in which the crown and abutment fractured as a unified structure. These findings may suggest that in high-stiffness materials, stresses may be less effectively dissipated at the adhesive interface and may instead be transmitted to the abutment. In particular, for the EB, the combined effect of its high rigidity and stress concentration associated with the knife-edge configuration may have promoted greater stress transfer to the abutment rather than dissipation at the adhesive interface, potentially contributing to the catastrophic fractures involving the abutment observed in this study. Thus, although EB exhibited a high fracture load, its failure pattern was not necessarily favorable in terms of abutment preservation. These findings suggest that the effect of margin design on fracture behavior may depend on material properties and stress transmission characteristics.

For SM materials, HC did not exhibit a fracture load sufficiently exceeding the late mixed dentition occlusal force (433 N) [16] in any margin configuration. For CP, fracture loads exceeding the late mixed dentition occlusal force (433 N) [16] were observed in both the knife-edge and chamfer configurations, suggesting a higher static load-bearing capacity relative to the reported occlusal force under the conditions tested. In contrast, the deep chamfer configuration yielded fracture loads below this occlusal threshold, suggesting that a sufficient load-bearing capacity may not be achieved, depending on the marginal design. Accordingly, the knife-edge and chamfer configurations showed relatively higher fracture resistance for CP under the static in-vitro conditions evaluated in the present study. In contrast, EB and DT exhibited high fracture loads across all marginal configurations; however, catastrophic fractures involving the abutment were observed at failure in the knife-edge configuration. These findings further illustrate that a higher fracture load does not necessarily correspond to a less severe failure pattern or greater protection of the abutment. This behavior may be attributable to their high stiffness and stress transmission characteristics, whereby stresses may be less effectively dissipated at the margin and instead transferred to the abutment. Therefore, under the conditions evaluated in the present study, the chamfer margin configuration may provide a more favorable fracture pattern for these materials from a biomechanical perspective, potentially reducing excessive stress concentration on the abutment. The ND did not reach the occlusal force of the late mixed dentition [16]; however, it exceeded the occlusal force of the early deciduous dentition [16] under certain conditions. Although the effect of the marginal configuration on improving the fracture load was limited, the observed fracture modes were relatively mild and less invasive to the abutment. These findings are consistent with previous reports, indicating limitations in the mechanical load and durability of additively manufactured (AM) prostheses [43]. Collectively, these results suggest that while HC and ND may provide sufficient functional performance in early stage occlusal environments during growth, their long-term use is restricted, supporting their use as provisional restorative materials.

Within the limitations of this study, the interaction between material properties and marginal configuration appears to play a critical role in determining the fracture behavior of CAD-CAM crowns for deciduous teeth. Accordingly, clinical decision-making should consider not only the fracture load but also the fracture mode and potential impact on the abutment tooth.

Because deciduous teeth have unique anatomical and mechanical characteristics [44, 45], including thinner enamel and dentin [10, 45] and enamel with lower hardness and elastic modulus than permanent teeth (nanohardness: 3.37 GPa; elastic modulus: 81.78 GPa) [44], and are exposed to age-dependent occlusal forces [16], material selection should consider both fracture load and fracture behavior, as these characteristics make deciduous teeth more susceptible to fracture under external forces.

Accordingly, under the conditions evaluated in the present study, fracture load should not be considered the sole indicator of clinical performance. Both fracture resistance and fracture severity, including abutment involvement and the potential for repairable failure, should be considered as complementary endpoints when evaluating deciduous crowns.

This study had several limitations. First, as an in vitro investigation, it did not fully reproduce the oral environment, including saliva, temperature fluctuations, and the complex cyclic loading conditions associated with mastication. Second, the fracture resistance was evaluated using a static load-to-fracture test; however, in clinical settings, restorations are subjected to fatigue loading. Therefore, further studies that incorporate fatigue testing and thermal cycling are required. Third, a standardized short-fiber-reinforced composite resin (everX Flow) was used as the abutment substrate rather than natural deciduous teeth. Although this approach reduced variability in morphology and mechanical properties, the homogeneous structure of the artificial abutment does not reproduce the enamel, dentin, and enamel–dentin junction of natural deciduous teeth. Consequently, the initiation and propagation of cracks and the resulting abutment fracture patterns may differ from those occurring in natural teeth. Fourth, all AM crowns were fabricated using a single, fixed build orientation perpendicular to the loading direction. Because additively manufactured resin materials may exhibit orientation-dependent mechanical behavior due to their layered structure and interlayer bonding, the fracture behavior observed in the present study may be specific to the selected build orientation and printing protocol. Therefore, these findings cannot necessarily be generalized to crowns fabricated using different build orientations or printing parameters. Fifth, although fracture modes were classified by two examiners who were blinded to the material and tooth preparation design, interobserver reliability was not assessed.

Future studies should assess long-term performance, together with fatigue loading, thermal cycling, and water storage, and should evaluate different build orientations and natural deciduous tooth substrates.

## Conclusion

In pediatric dentistry, crown selection should consider both fracture load and fracture behavior, including abutment involvement. The influence of margin design on fracture behavior varied depending on the crown material. In subtractive-manufacturing materials, differences in fracture load were observed alongside differences in the reported mechanical properties, whereas internal structure appeared to contribute to differences in fracture behavior among additive-manufacturing materials. CP showed relatively higher fracture resistance with knife-edge and chamfer margins under the static in-vitro conditions evaluated in this study. Although EB and DT exhibited high fracture loads, knife-edge margins were associated with catastrophic abutment fractures, whereas chamfer margins showed more favorable fracture patterns under the present experimental conditions. In contrast, HC and ND showed lower fracture loads but predominantly repairable fracture patterns, which may indicate a less severe failure pattern under the present experimental conditions. These findings indicate that fracture load should not be considered the sole indicator of performance and that fracture severity and abutment involvement should be evaluated as complementary endpoints.

## Data Availability

The datasets generated and analyzed during the current study are available from the corresponding author on reasonable request.
